# Preoperative circulating peroxiredoxin 1 levels as a predictor of non-alcoholic fatty liver disease remission after laparoscopic bariatric surgery

**DOI:** 10.3389/fendo.2022.1072513

**Published:** 2022-12-21

**Authors:** Xiaoyun Cheng, Zhibing Fu, Wei Xie, Liyong Zhu, Jie Meng

**Affiliations:** ^1^ Department of Pulmonary and Critical Care Medicine, The Third Xiangya Hospital of Central South University, Changsha, Hunan, China; ^2^ Department of Pulmonary and Critical Care Medicine, Xiangya Hospital of Central South University, Changsha, Hunan, China; ^3^ Hunan Key Laboratory of Organ Fibrosis, Central South University, Changsha, Hunan, China; ^4^ Department of General Surgery, The Third Xiangya Hospital, Central South University, Changsha, China; ^5^ Department of Cardiology, Xiangya Hospital, Central South University, Changsha, China

**Keywords:** non-alcoholic fatty liver disease, insulin resistance, obesity, peroxiredoxin 1, laparoscopy sleeve gastrectomy, nomogram

## Abstract

**Background:**

Non-alcoholic fatty liver disease (NAFLD) is associated with obesity and insulin resistance and can be improved after bariatric surgery. Circulating Peroxiredoxin 1 (Prdx1) protein was reported to regulate energy metabolism and inflammation. This study aimed to investigate the roles of serum prdx1 in NAFLD patients with obesity undergoing LSG and to develop a prognostic model to predict the remission of severe NAFLD.

**Methods:**

The data of 93 participants from a tertiary hospital were assessed. Before laparoscopic sleeve gastrectomy (LSG) and three months after LSG, anthropometric parameters, laboratory biochemical data, and abdominal B-ultrasound results were collected, and their hepatic steatosis index (HSI) and triglyceride-glucose index (TyG) were calculated. A NAFLD improvement (NAFLD-I) nomogram prediction model was constructed using the least absolute shrinkage and selection operator (LASSO) regression and multiple regression, and its predictive ability was verified in a validation cohort.

**Results:**

The baseline Prdx1 (OR: 0.887, 95% CI: 0.816-0.963, *p*=0.004), preoperative TyG (OR: 8.207, 95% CI: 1.903-35.394, *p*=0.005) and HSI (OR: 0.861, 95% CI: 0.765-0.969, *p*=0.013) levels were independently associated with NAFLD-I at three months after LSG in NAFLD patients with obesity. In the primary and validation cohorts, the area under the receiver operating characteristic (AUC) of the developed nomogram model was 0.891 and 0.878, respectively. The preoperative circulating Prdx1 levels of NAFLD patients with obesity were significantly reduced after LSG (25.32 [18.99-30.88] *vs*. 23.34 [15.86-26.42], p=0.001). Prdx1 was related to obesity and hepatic steatosis based on correlation analysis.

**Conclusion:**

The nomogram based on preoperative serum prdx1, HSI and TyG could be an effective tool for predicting remission of severe NAFLD after LSG.

## 1 Introduction

In recent years, the prevalence of non-alcoholic fatty liver disease (NAFLD) has increased significantly (1). More worryingly, a data framework predicted that a growing number of NAFLD patients would develop non-alcoholic steatohepatitis (NASH)-related end-stage or malignant liver diseases (2), who have become the main population for liver transplantation. The aggravating incidence of NAFLD and the severe consequences of disease progression makes it necessary to develop effective methods to predict NAFLD response to LSG treatment. In this regard, serological biomarkers and models have attracted significant clinical attention due to their non-invasive, low cost, simple maneuverability and strong repeatability advantages.

Hepatic steatosis, inflammation and fibrosis in NAFLD are closely related to obesity and insulin resistance (IR) (3, 4). Accumulating evidence suggests that the Peroxiredoxin (PRDX) family is a key antioxidant enzyme regulating the balance between glucose and lipid metabolism (5). PRDX members, including Prdx1 (6), Prdx2 (7), Prdx3 (8), Prdx4 (9) and Prdx6 (10, 11), were reported to be overexpressed in obesity, type 2 diabetes mellitus (T2DM) and atherosclerosis (12). Prdx1 and Prdx4 are the secretory members of the PRDX family. Elevated circulating Prdx4 was found to be associated with certain components (e.g., hypertension and triglycerides [TG]) and mature inflammatory markers (e.g., high-sensitivity C-reactive protein [hs-CRP] and procalcitonin) of the metabolic syndrome (13) and has been incorporative in a T2DM DESIR model in men (14).

vPrdx1 is the main PRDX family member in the pancreas (15) (16), liver insulin resistance, inflammation and steatosis (17). An increase in Prdx1 is released through exosomes in response to various environmental stressors (18), such as inflammatory cytokines (19), oxidative stress (20) and streptozotocin (STZ) administration (21). At present, two studies have reported an increase in Prdx1 in T2DM was positively correlated with the homeostasis model assessment of insulin resistance (HOMA-IR) (r = 0.276, p< 0.01) (18, 22). However, the level and change of circulating Prdx1 in the evolution of NAFLD have not yet been reported.

Metabolic surgery is the most effective treatment to achieve substantial and lasting weight loss in individuals with obesity (23). Among patients with severe obesity undergoing metabolic surgery, the prevalence of NAFLD was found to exceed 90%, the prevalence of NASH was reported to approximate 37% (24 to 98%), with up to 5% of the patients at high risk for unanticipated liver cirrhosis (24). After bariatric surgery, visceral fat and insulin resistance are reduced within a few days (25), liver fatty acid uptake is reversed (26), and liver inflammation is improved (27, 28). LSG has replaced the Roux-en-Y gastric bypass surgery (RYGB) as the most common bariatric operation (29). In particular, NAFLD individuals benefit more from LSG than RYGB (30), with a reported -2.5 (95% CI: -3.5 to -1.5) median NAFLD activity score (NAS) improvement at 192 days after LSG (31).

Since circulating Prdx1 is closely related to chronic metabolism diseases (i.e., arteriosclerosis, diabetes, etc.) and inflammation, we hypothesized that increased circulating Prdx1 might be a biomarker of obesity, insulin resistance and NAFLD and therefore played an important role in the clinical response after LSG.

## 2 Methods

### 2.1 Study design and patients

**Figure 1:**
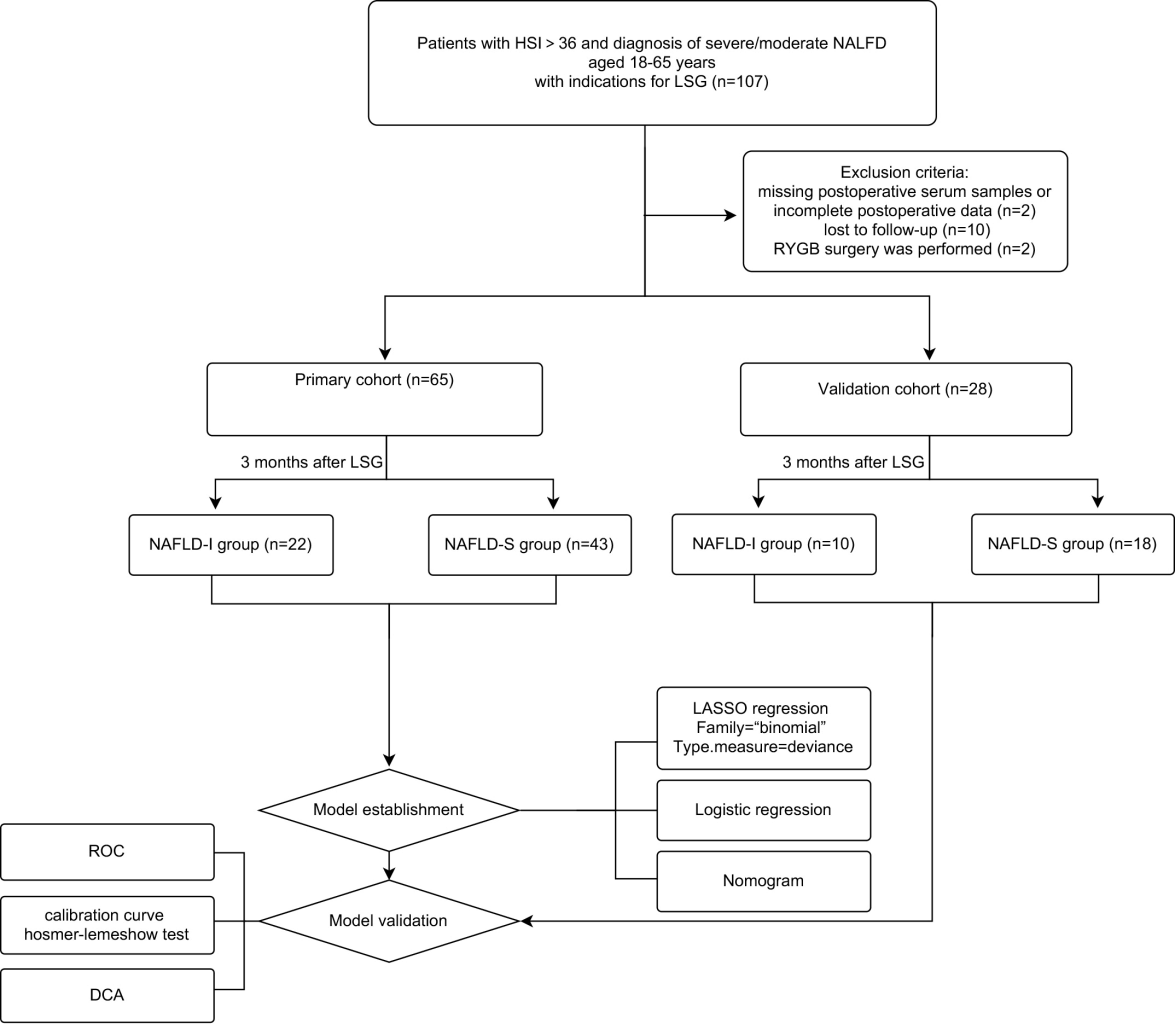
Flow diagram of study design. NAFLD, non-alcoholic fatty liver disease; NAFLD-I, NAFLD improvement (postoperative HSI<36 and without diagnostic fatty liver by ultrasound); NAFLD-S, NAFLD sustain; LSG, laparoscopic sleeve gastrectomy; RYGB, Roux-en-Y gastric bypass surgery; LASSO, least absolute shrinkage, and selection operator; ROC, receiver operating characteristic curve; DCA, decision curve analysis.

The study design was a prospective observational study conducted at a single center, as shown in **Figure 1**. All subjects gave their informed consent for inclusion before participating in the study. The study was conducted in conformity with the Declaration of Helsinki, and the protocol was approved by the Ethics Committee of the Third Xiangya Hospital (Project identification code: R20047). According to the indications and contraindications of LSG guidelines (32), from April 2019 to October 2019, NAFLD patients who underwent LGS in the Department of General Surgery, the Third Xiangya Hospital, were consecutively enrolled. Subjects met all the following inclusion criteria: (1) with the hepatic steatosis index (HSI)>36 and preoperative diagnosis of severe/moderate NAFLD by B-ultrasound; (2) individuals with obesity that combined with other comorbidities of metabolic syndrome; (3) aged 18 to 65 years old. The exclusion criteria were: (1) with the contraindications of LSG; (2) severe infection or organic diseases, such as myocardial infarction, renal failure, and stroke; (3) long-term use of liver injury-inducing drugs, alcoholism, or drug addiction; (4) previous metabolic surgery; (5) missing serum samples, incomplete data, or loss to follow-up. In accordance with previous reports, the LSG was performed by the same medical team (33). Finally, a total of 93 patients were enrolled and randomly divided into primary cohort (n=65) and validation cohort (n=28) at 7:3. During the same period, fifty-seven healthy adult controls were recruited at the health examination center of the same Hospital. The inclusion criteria were adults with a BMI of 18.5 to 24.0 kg/m2. Exclusion criteria were acute or chronic systemic disease, pharmacological treatment, alcohol abuse, history of abdominal surgery, and absence of informed consent.

### 2.2 The data, calculated index and definition

Anthropometric parameters, laboratory biochemical data, and abdominal B-ultrasound results were collected before LSG and three months after LSG. Circulating PRDX1 levels were measured using a commercially available enzyme-linked immunosorbent assay (ELISA) kit (KA0536, Abnova, Taiwan, China) in the serum of isolated blood samples collected from the patients. Preoperative fasting serum was assessed to measure PRDX1 concentration.

The anthropometric parameters included height, weight, waist circumference, and hip circumference. Laboratory results included white blood cell (WBC) count, neutrophil to lymphocyte ratio (NLR), platelet count, fasting blood glucose (FBS), fasting insulin (FINS), C-peptide level, triglyceride (TG), total cholesterol (TC), low-density lipoprotein (LDL), high-density lipoprotein (HDL); alanine aminotransferase (ALT), aspartate aminotransferase (AST), albumin (ALB), globulin (GLB), blood urea nitrogen (BUN), creatinine and uric acid (UA). For the imaging examination, abdominal B-ultrasound was performed by two experienced radiologists using the Diagnostic Ultrasound System and Transducers (Philips EPIQ7C, N.V., Amsterdam, the Netherlands). Body mass index (BMI), HSI, non-alcoholic fatty liver disease fibrosis score (NFS), fibrosis 4 score (F4), HOMA-IR, quantitative insulin sensitivity check index (QUICKI), triglyceride-glucose index (TyG), the percentage of total body weight loss (%TBWL), and percentage of excess weight loss (%EWL) were calculated. The formula of each index and their threshold in the Chinese population are shown in **Table 1**.

**Table 1:** The formula and threshold of calculated index.

Index		Formula	Threshold	References
NAFLD				
HSI		*HSI* = 8 × (*ALT/AST*) + *BMI* (+2, *if female*; +2 *if diabetes*)	>36	(34)
NFS		$\begin{array}{l} NFS=1.675+(0.037\times age)+(0.094\times BMI)+\\ (1.13\;\times \;diabetes\;[YES\;=\;1,NO\;=\;0])\;+\;\left(0.99\;\times \;\frac{AST}{ALT}\right)-\\ \left(0.013\times PLT)\;\times \;(0.66\;\times \;albumin\right) \end{array}$	<-1.455:F0-F2: -1.455 to 0.675: Uncertain >0.675: F3-F4	(34)
F4		*F*4 = (*age* × *AST*)/(*PLT* × √ *ALT*)	<1.3: F0-F2: <2.67: F3-F4	(35)
IR				
HOMA-IR		*HOMA-IR* = *FPG* × *FINS*/22.5	<2.69	(36)
QUICKI		*QUICKI* = 1/(*Log FPG* + *Log FINS*)	≤0.339	(37)
TyG		*TyG* = *Ln* [*TG* × *FINS*/2]	Male>8.81,Female> 8.73	(38)
Weight loss				
BMI	*BMI* = *weight/height* ^˄^2		≥ 28 kg/m^2^: obesity	(39)
%TBWL	%*TBWL* = [(*initial weight*)- (*postop weight*)]/[(*initial weight*)] × 100)		-	(40)
%EWL	%EWL = (initial weight – postoperative weight)/(initial weight – ideal weight for BMI of 25 × 100		-	(29)

HSI, hepatic steatosis index; NFS, non-alcoholic fatty liver disease fibrosis score; F4, fibrosis 4 score, HOMA-IR, the homeostasis model assessment of insulin resistance; QUICKI, quantitative insulin sensitivity check index; TyG, triglyceride-glucose index; BMI, body mass index; %TBWL, percentage total body weight loss; %EWL, percentage excess weight loss.

Age: year; PLT: ×10^9; albumin: g/dL; AST and ALT: U/L; FPG: mmol/L; FINS: μU/mL; TG: mg/dL; FINS: mg/dl; weight: kg; height: m.

HSI has been validated by ultrasonography and magnetic resonance imaging (MRI) and is widely used in clinical studies of metabolic diseases (3, 41). A specificity of 92% was reported for diagnosing NAFLD in patients with HSI >36 (42), and the same cut-off value was thereby adopted in our study. NAFLD improvement (NAFLD-I) was referred to as HSI<36 at three months after LSG, with no fatty liver diagnosed by postoperative ultrasound; otherwise, NAFLD sustain (NAFLD-S) was considered.

### 2.3 Statistical analysis

SPSS v19 (IBM, Armonk, NY) and GraphPad Prism (GraphPad, San Diego, California) were used for statistical analyses and to draw indicated plots. Enumeration data are shown as n (%) and were compared with the chi-square tests. All measurement data are expressed in median (25th–75th percentiles [Q1–Q3]) and were analyzed using nonparametric tests. The Wilcoxon paired rank test was used for preoperative and postoperative group comparisons. A *p*-value< 0.05 was considered statistically significant.

The data were further processed with R studio (version 4.2.0; R studio, Boston, Massachusetts). Briefly, we installed the “glmnet” package to perform LASSO regression in the primary cohort to select the preoperative variables with the strongest influence on NAFLD-I. Factors with *p*< 0.05 in multivariate logistics regression were considered independent influencing factors of NAFLD-I and were used to develop our predictive nomogram model using the “rms” package.

In addition, the discrimination (receiver operator characteristic curve [ROC] by “pROC” package), calibration (calibration curve and Hosmer–Lemeshow test by “rms” and “ResourceSelection” package) and clinical utility (decision curve analysis [DCA] by “ggDCA” package) of our constructed nomogram were verified both in the primary cohort and the validation cohort. Lastly, the Spearman correlation coefficient was used to analyze the associations between preoperative parameters.

## 3 Results

### 3.1 Characteristics of the NAFLD at baseline and surgical outcomes

A total of 93 NAFLD patients were enrolled (median age: 30 years [inter-quartile range: 24-34]), comprising 29 (31.18%) males and 64 (68.82) females. The median body weight was 100.30 (90.65-118.15) kg, and the median BMI was 37.16 (34.17-42.70) kg/m^2^. Regarding comorbidities, 33.33% of the patients were diagnosed with T2DM before surgery. Three months after LSG, obesity, low-grade inflammation, insulin resistance, hepatic steatosis, hyperlipidemia and hyperuricemia were found to be significantly alleviated compared with before LSG (**Table 2**). Postoperative abdominal B-ultrasonography showed no, mild, moderate and severe NAFLD in 36 (38.71%), 22 (23.66%), 27 (29.03%) and 8 (8.60%) cases, respectively. The patients’ BMI, body weight and hip circumference were significantly reduced (*p*<0.001). TBWL% and EWL% were 18.33 (13.57-23.62) and 25.28 (18.76-32.23), respectively. We also observed a decrease in WBC count, NE% and NLR (*p*<0.001). Postoperative HOMA-IR, TyG and HSI were significantly lower than before the operation (*p*<0.001). Statistically significant differences were observed between post- and pre-surgical levels of ALT, AST, TG, UA (*p*<0.001), TC (*p*=0.015) and HDL (*p*=0.046).

**Table 2:** The analysis of preoperative and postoperative indicators by the Wilcoxon paired rank test tests.

	Pre-operation (n=93)	Post-operation (n=93)	Z value	*p* value
HSI	51.23(46.56-58.49)	39.89 (35.21-46.09)	-8.228	0.000**
BMI	37.16 (34.17-42.70)	29.71(26.03-34.82)	-7.604	0.000**
Weight	100.30 (90.65-118.15)	80.00 (69.90-97.20)	-7.494	0.000**
Waistline	114.00 (104.00-128.00)	105.50 (88.00-141.00)	-1.444	0.149
Hip circumference	119.00 (112.00-127.00)	104.00 (94.00-113.00)	-7.747	0.000**
WBC	7.73 (6.42-8.99)	6.64 (5.62-7.95)	-5.304	0.000**
NE%	60.25 (56.10-66.43)	56.70 (50.90-61.80)	-4.062	0.000**
LY%	29.75 (25.00-35.23)	34.60 (29.60-39.40)	-4.728	0.000**
NLR	2.01 (1.62-2.65)	1.63 (1.27-2.08)	-4.507	0.000**
Platelet	285.00 (242.50-325.50)	268.00 (237.75-319.00)	-2.060	0.039*
HOMA-IR	6.71 (4.86-12.80)	2.98 (1.93-4.27)	-8.236	0.000**
QUICKI	0.46 (0.41-0.49)	1.47 (1.31-1.69)	-8.339	0.000**
TyG	8.93 (8.64-9.58)	8.58 (8.28-8.88)	-5.977	0.000**
ALT	40.00 (26.00-68.00)	16.00 (12.00-26.50)	-8.073	0.000**
AST	28.00 (21.00-40.00)	18.00 (15.00-24.50)	-7.153	0.000**
TG	1.67 (1.30-2.47)	1.35 (0.99-1.71)	-5.363	0.000**
TC	5.00 (4.40-5.64)	4.43 (4.04-5.38)	-2.445	0.015*
LDL	2.96 (2.29-3.49)	2.99 (2.55-3.62)	-1.496	0.135
HDL	1.07 (0.97-1.17)	1.03 (2.55-3.62)	-1.993	0.046*
UA	445.00 (368.00-506.00)	379.00 (322.00-444.00)	-4.832	0.000**
NFS	-1.73 (-2.47-0.00)	-1.89 (-2.76- -0.62)	-1.316	0.188
F4	0.48 (0.35-0.63)	0.51 (0.35-0.67)	-5.121	0.609

*p<0.05, **p<0.001.

HSI, hepatic steatosis index; BMI, body mass index; WBC, white blood cell; NE%, neutrophil percentage; LY%, lymphocytes percentage; NLR, lymphocyte ratio; HOMA-IR, the homeostasis model assessment of insulin resistance; QUICKI, quantitative insulin sensitivity check index; TyG, triglyceride-glucose index; ALT, alanine transaminase; AST, aspartate transaminase; TG, triglyceride; TC, total cholesterol; LDL, low-density lipoprotein; HDL, high-density lipoprotein; UA, uric acid; NFS, non-alcoholic fatty liver disease fibrosis score; F4, fibrosis 4 score.

Further analysis showed no significant differences in the baseline age, gender, degree of obesity, insulin resistance, hepatic steatosis and circulating Prdx1 levels between the primary cohort and the validation cohort (*p* > 0.05) (**Table 3**), suggesting that both cohorts were homogeneous.

**Table 3:** Preoperative variables between the primary cohort, the validation cohort, and the control cohort.

	Primary cohort (n=65)	Validation cohort (n=28)	*p* value	Control cohort (n=57)
Age (year)	28 (24-33.5)	32 (24-37.75)	0.261	32 (23.5-41.5)
Male (%)	20 (30.77)	9 (32.14)	0.896	22 (38.60)
T2DM (%)	21(32.31)	10(35.71)	0.749	0
NAFLD-I (%)	22(33.8)	10 (35.71)	0.862	–
BMI	36.88(34.27-42.99)	37.75(33.20-41.52)	0.821	21.49(20.19-23.32)
Weight	100.30(90.30-122.20)	100.30(92.23-112.10)	0.666	57.80 (53.40-62.50)
HOMA-IR	7.22(5.20-12.80)	6.12(3.76-13.68)	0.197	2.39(2.19-2.77)
QUICKI	0.45(0.41-0.48)	0.47(0.40-0.52)	0.197	0.33(0.32-0.34)
TyG	8.93(8.55-9.57)	8.91(8.76-9.64)	0.431	7.92(7.64-8.14)
HSI	51.23(41.56-58.94)	51.34(46.17-56.53)	0.776	29.08(27.48-31.60)
Prdx1	25.32(17.75-34.01)	26.60(18.89-32.02)	0.688	12.38 (8.370-15.71)

T2DM, diabetes mellitus type 2; NAFLD-I, hepatic steatosis index improvement; BMI, body mass index; HOMA-IR, the homeostasis model assessment of insulin resistance; QUICKI, quantitative insulin sensitivity check index; TyG, triglyceride-glucose index.

### 3.2 Preoperative and postoperative circulating Prdx1 Levels in NAFLD patients with obesity

**Figure 2:**
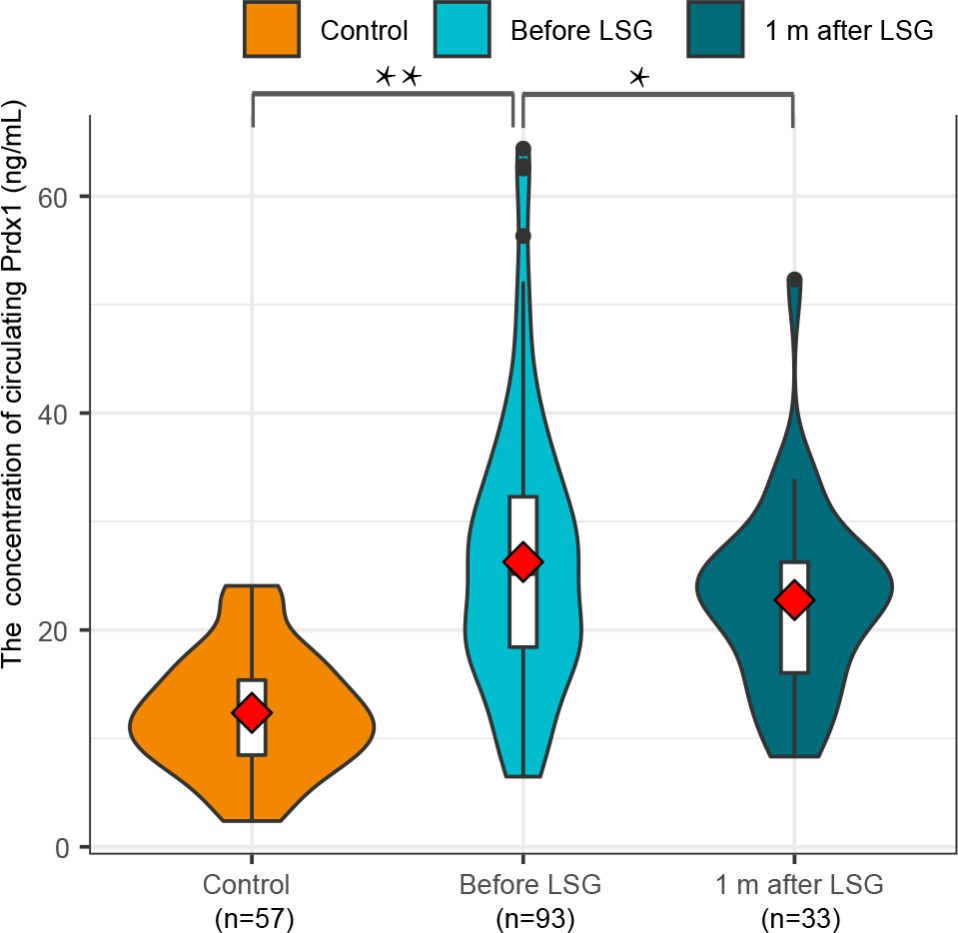
A comparison of circulating Prdx1 levels in NAFLD patients with obesity before and after surgery.

The median age of the normal control group was 32 (23.5-41.5) years and comprised 22 (38.60%) males and 35 (61.40%) females. The participants in the Control cohort’s median BMI was 21.49 (20.19-23.32) kg/m^2^ and had no history of T2DM or NAFLD. Their weight, HOMA-IR, QUICKI, TyG and HSI are shown in **Table 3**. The Control group was comparable with the NAFLD group in terms of age (Z=-1.556, p=0.120) and sex (χ2 = 0.866, p=0.352). Preoperative Prdx1 (ng/mL) was significantly higher in NAFLD patients with obesity than in normal controls (25.51 [18.51-32.83] *vs*. 12.38 [8.370-15.71], p<0.001). Postsurgical serum collection was conducted one month after surgery in 33 patients. The Wilcoxon paired rank tests showed that patients after LSG had a lower concentration of Prdx1 than those before surgery (25.32 [18.99-30.88] *vs*. 23.34 [15.86-26.42], p=0.001) (**Figure 2**).

### 3.3 Identifying independent influencing factors

**Figure 3:**
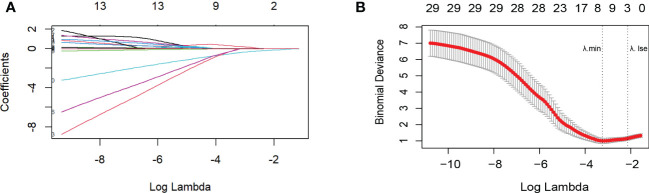
Variable selection by the LASSO binary logistic regression model. **(A)** The plot of the coefficient profile against log(lambda). As lambda reduced, the compression parameter decreased, and the absolute value of both coefficients increased. **(B)** Three factors with regression coefficients greater than 0 were selected (lambda value between lambda.min and lambda.1se). The optimized lambda value was between lambda.min and lambda.1se, indicating that three factors with regression coefficients greater than zero were selected.

The Lasso analysis identified three predictors with non-zero regression coefficients (**Figures 3A, B**). In multivariate logistic regression analysis, we found that preoperative TyG (OR = 8.207, 95% CI = 1.903-35.394, *p*=0.005), HSI (OR = 0.861, 95% CI = 0.765-0.969, *p*=0.013) and circulating Prdx1 (OR = 0.887, 95% CI = 0.816-0.963, *p*=0.004) were independently associated with NAFLD remission at three months after LSG (**Table 4**).

**Table 4:** Multivariate logistic regression of NAFLD-I (n=65).

Variables	B	S.E.	Wals	*p*	Exp(B)	95%CI for Exp(B)	
						(2.5%)	(97.5%)
TyG	2.105	0.746	7.969	0.005	8.207	1.903	35.394
Circulating Prdx1	-0.120	0.042	8.075	0.004	0.887	0.816	0.963
HSI	-0.150	0.061	6.125	0.013	0.861	0.765	0.969

NAFLD-I, non-alcoholic fatty liver disease improvement; TyG, triglyceride-glucose index; Prdx1, Peroxiredoxin 1.

### 3.4 Predictive model construction, validation and evaluation

**Figure 4:**
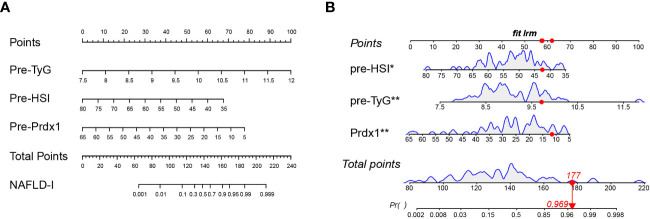
Nomogram prediction model. **(A)** TyG, HSI, and Prdx1 as risk factors of the model. **(B)** Example of a dynamic nomogram. For the patient with preoperative HSI of 42.53, TyG of 9.71, and prdx1 concentration of 11.55 ng/mL, his/her total score in this nomogram model was 177 points, and the corresponding probability of NAFLD remission at three months after LSG was predicted to be 96.9%. NAFLD-I, non-alcoholic fatty liver disease improvement; TyG, triglyceride-glucose index; Prdx1, Peroxiredoxin 1.

**Figure 5:**
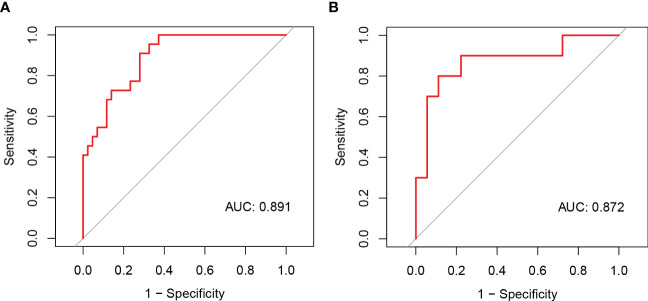
The ROC curves of the nomogram model of NAFLD remission at three months after LSG in the primary **(A)** and validation cohorts **(B)**.

**Figure 6:**
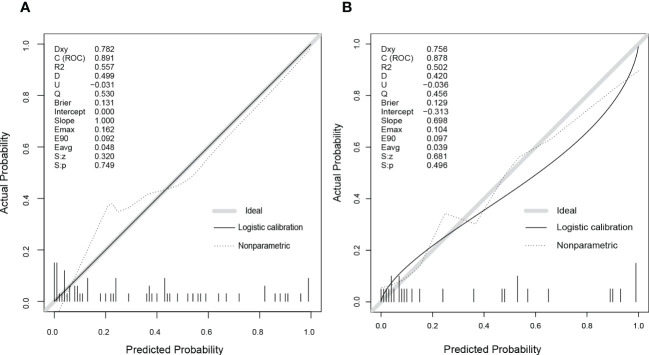
The calibration curves of the nomogram model in the primary **(A)** and validation cohorts **(B)**. As shown on graphs, the X-axis represented the predicting probability of postoperative NAFLD remission, and the Y-axis illustrated the actual outcomes in follow-up. The diagonal grey line was the perfect prediction made by the ideal model, the solid black line was the performance by nomogram, and the dashed line was the corrected model performance. As the model performance approached the diagonal grey line, the nomogram’s prediction accuracy increased.

**Figure 7:**
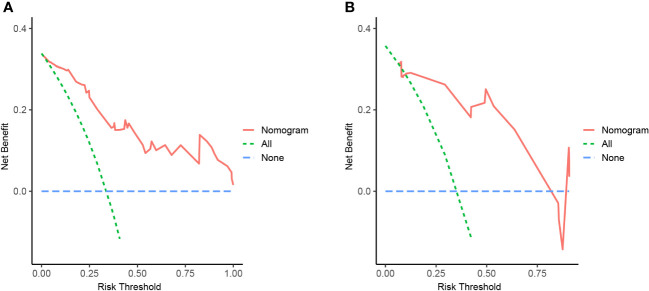
Nomogram of NAFLD remission at 3 months after LSG with decision curve analysis (DCA). The Y-axis showed net benefit. The blue line represented the assumption that none of NAFLD patients have been performed with LSG, while the green line represented the assumption that all patients have been performed with LSG, and the red line represented using this nomogram to predict NAFLD-I in patients. Clinical application value increased with increasing distance between red solid line and blue line. **(A)** from the training set, **(B)** from the validation set.

We developed a Lasso-Logistic nomogram model to assess the independent factors associated with NAFLD remission three months after LSG (**Figures 4A, B**). Then, ROC curves were used to determine the discrimination of this prediction model. Based on the results, our nomogram demonstrated high predictive values (area under the receiver operating characteristic [AUC] for the primary cohort: 0.891, 95% CI: 0.816-0.966; AUC for validation cohort: 0.878, 95% CI: 0.716-1.000) (**Figures 5A, B**). Additionally, findings from the calibration graph (**Figures 6A, B**) and Hosmer-Lemeshow test showed that our constructed nomogram was highly consistent with the actual observation (Hosmer-Lemeshow test in primary cohort: *p*=0.495; validation cohort: *p*=0.138), and the clinical utility of the nomogram model was assessed *via* DCA in both the primary and validation cohorts (**Figures 7A, B**), which showed that the application of the nomogram model had higher net benefit than “treat all NAFLD patients with LSG” and “treat none of NAFLD patients with LSG” when the risk threshold was between 0.5% and 99.8% in the primary cohort and between 5.4% and 83.3% in the validation cohort.

### 3.5 Correlation analysis

**Figure 8:**
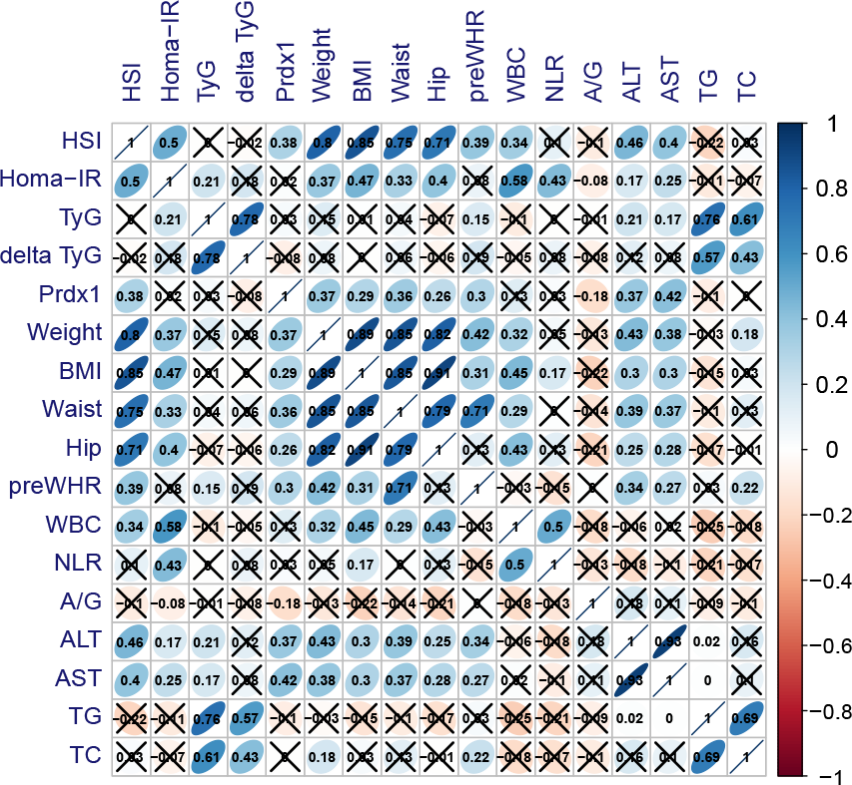
Association between baseline indications and postoperative Prdx1. Blue presented highly positive correlation coefficients, whereas red presented highly negative correlation coefficients. HSI, hepatic steatosis index; TyG, triglyceride-glucose index; delta TyG, the change of TyG was quantified by subtracting preoperative values from postoperative values; Prdx1, Peroxiredoxin 1; BMI, body mass index; WHR, waist-to-hip ratio; WBC, white blood cells; NLR, neutrophil to lymphocyte ratio; A/G, albumin/globulin; ALT, alanine transaminase; AST, aspartate transaminase; TG, triglyceride; TC, total cholesterol.

Correlation analysis showed that HSI was positively correlated with HOMA-IR (r=0.530, *p*<0.001), Prdx1 (r=0.383, *p*=0.002) and body weight (r=0.799, *p*<0.001) (**Figure 8**). Additionally, Prdx1 was positively correlated with hepatic steatosis, obesity, ALT and AST.

## 4 Discussion

The most common bariatric procedures are sleeve gastrectomy (SG) and Roux-en-Y gastric bypass (RYGB), which are both effective in alleviating hepatic steatosis, while SG was reported to be associated with fewer complications (43). A published study (44) investigating liver fat fraction at one-year follow-up after LSG found a reduction of -19.7% (95% CI: -22.5% to -16.9%). Most studies about NAFLD have focused on the efficacy of LSG after one year or longer. In this present study, we assessed the remission of NAFLD at three months after LSG because it was at that time period that we observed improvements in relevant pathological status (i.e., insulin resistance, obesity) and liver biopsy after surgery. Studies have shown that within three months after bariatric surgery, insulin resistance usually returns to a normal level (45), with significant weight loss achieved in patients with obesity (46). A reduction in hepatic fat content and improvement in hepatic insulin resistance are considered the earliest beneficial effects of bariatric surgery (47). In this study, liver biopsies were performed to confirm that hepatic steatosis, fibrosis and NAFLD activity scores decreased three months after surgery (48). In addition, we also found an early remission of NAFLD after LSG could be accurately predicted using the proposed nomogram based on the patients’ preoperative TyG, HSI and Prdx1 concentration.

A previous study reported that preoperative steatosis (*p* = 0.006) and insulin resistance index (*p* = 0.010) were independent predictors of postsurgical persistent severe steatosis one year after surgery *via* liver biopsy (49). However, the wide application of serial liver biopsy is limited due to its invasiveness, variability in sample collection, and inconsistent evaluation. In terms of imaging exams, MRI is not routinely performed in NAFLD as it is time-consuming and expensive. A meta-analysis indicated that B-ultrasound and transient elastography failed to identify and stage NAFLD in individuals with high BMI (50). Comparatively, a serological model could demonstrate high accuracy in diagnosing hepatic steatosis. HSI is composed of BMI, DM and ALT/AST ratio. Encouragingly, a previous study showed that its AUC for diagnosing NAFLD was 0.81 in the training cohort and 0.82 in the validation cohort (51). More importantly, our study suggested that HSI not only had a good performance in diagnosing NAFLD but was also an important independent predictor of early NAFLD remission after LSG (OR: 0.861, 95% CI: 0.765-0.969, *p*=0.013). Our correlation analysis of preoperative indicators showed that preoperative TyG was significantly associated with insulin resistance and obesity, consistent with a previous report (52). Interestingly, we also found that HSI was positively correlated with circulating Prdx1 (r=0.383, *p* =0.002). Several studies have observed increased inflammatory proteins, such as C-reactive protein, which declined after bariatric surgery in T2DM patients with obesity (53).

Circulating PRDX1 was recently identified as a damage-associated molecular pattern (DAMP) that acted as a proinflammatory factor by activating the Toll-like receptor 4-mediated signaling (54). In chronic inflammation, more than a quarter of Prdx1 was secreted actively to the outside of cells as exosomes (55, 56) or through a mechanism of redox-dependent secretion (57). Cross-sectional studies showed that Prdx1 increased in chronic metabolic diseases (i.e., atherosclerosis, diabetes, etc.). However, there is limited research on Prdx1 in the evolution of NAFLD. Our study showed for the first time a high circulating Prdx1 concentration in NAFLD patients with obesity and that its level decreased one month after LSG. Moreover, high baseline Prdx1 levels were associated with poor remission of NAFLD three months after LSG (OR: 0.887, 95% CI: 0.816-0.963, *p*=0.004). We also observed that Prdx1 plasma concentration positively correlated with BMI, HSI, AST and ALT. Given these observations, combined with recent evidence which showed that the overexpression of Prdx1 induced by oxidative stress enhanced insulin resistance and the accumulation of lipid droplets in mouse’s HepG2 cells (6), we speculated that Prdx1 might be involved in inflammation, oxidative stress and liver fat deposition in NAFLD patients with obesity. However, this hypothesis could not be confirmed in this present study, and additional studies are needed for further clarification.

Of the complex factors in NAFLD (obesity, lipid metabolism disorders, genes, etc.), insulin resistance is the one that has changed most dramatically within one week after LSG. After one week following SG, HOMA-IR reduced significantly from 6.7 ± 11 to 3.0 ± 1.6 (*p* = 0.019) (3). In addition, a study that reported improved NAFLD with a low-calorie ketogenic diet (VLCKD) found that baseline insulin resistance was a positive independent predictor (r=0.414, *p*=0.009) of HSI reduction (58). Comparatively, a study of 4784 Chinese adults reported that TyG was a better predictor for NAFLD when compared with HOMA-IR (AUC=0.761) (59). Furthermore, we found that higher TyG was more likely to lead to NAFLD remission, suggesting that excessive insulin resistance might be strongly recommended as an indication of LSG intervention in NAFLD. The most likely reason was that insulin resistance was more significantly reversed in patients with higher baseline TyG, based on our correlation analysis, in which pre-surgical TyG showed a strong positive correlation with delta TyG (r=0.78, *p*< 0.001). Similarly, two five-year prospective studies found that patients with refractory insulin resistance had persistent obesity and steatosis after surgery (60, 61).

According to our findings, baseline TyG was an independent predictor of HSI remission at three months after LSG in NAFLD patients with obesity, and it is the first time that circulating Prdx1 is proposed as a promising biomarker to assess postoperative improvement of obesity and NAFLD. Significant insulin resistance remissions are more likely to occur in patients with higher TyG.

Despite the interesting and novel findings reported, our study had some limitations. First, the sample size was limited, even though we internally validated the predictive ability of the nomogram *via* bootstrapping (**Supplementary Figure 1**), and compared it with another prediction model which was built (**Supplementary Figures 2**, **3A**) and validated (**Supplementary Figures 3B–D**) in all samples (n=93). Second, we used B-ultrasound results and surrogate parameters as a standard instead of histology in this study; thus, our prediction results might not perfectly match NAFLD remission assessed by pathological criteria. In addition, NASH is a severe complication of NAFLD, which might also be associated with fibrosis, cirrhosis and hepatocellular carcinoma. We could not assess the different pathological prognoses of NAFLD (non-alcoholic fatty liver and NASH) due to a lack of liver histology data in this study, even though HSI and ultrasonography were externally validated against liver biopsy or magnetic resonance spectroscopy (62).

In summary, TyG and Prdx1 were identified as important predictors of NAFLD remission, which could be used as an early non-invasive marker or as adjuncts to liver biopsy to confirm NAFLD remission. A future clinical follow-up study would provide further evidence for assessing the changes in TyG and Prdx1 over time in patients with NAFLD.

## Data availability statement

The raw data supporting the conclusions of this article will be made available by the authors, without undue reservation.

## Ethics statement

The studies involving human participants were reviewed and approved by the Ethics Committee of the Third Xiangya Hospital (Project identification code: R20047). The patients/participants provided their written informed consent to participate in this study.

## Author contributions

All authors contributed to the conception and design of the study. XC, JM and ZF organized the database. XC, ZF, WX and LZ performed the statistical analysis. All authors wrote the first draft of the manuscript. All authors wrote sections of the manuscript. All authors contributed to the article and approved the submitted version.
