# Supplementary material for: Preoperative circulating peroxiredoxin 1 levels as a predictor of non-alcoholic fatty liver disease remission after laparoscopic bariatric surgery
Source: Front Endocrinol (Lausanne). 2022 Dec 21;13:1072513. doi: 10.3389/fendo.2022.1072513 (PMC9810748; doi:10.3389/fendo.2022.1072513)
Supplement: Supplementary file 1 [file DataSheet_1.docx]

Supplementary Material

Preoperative circulating Peroxiredoxin 1 levels as a predictor of non-alcoholic fatty liver disease remission after laparoscopic bariatric surgery

**Xiaoyun Cheng^1,2,3†^, Zhibing Fu^4†^, Wei Xie^5^, Liyong Zhu^4*^, Jie Meng^1,3*^**

^1^Department of Pulmonary and Critical Care Medicine, The Third Xiangya Hospital of Central South University, Changsha, Hunan, China

^2^Department of Pulmonary and Critical Care Medicine, Xiangya Hospital of Central South University, Changsha, Hunan, China

^3^Hunan Key Laboratory of Organ Fibrosis, Central South University, Changsha, Hunan, China

^4^Department of General Surgery, The Third Xiangya Hospital, Central South University, Changsha, China

^5^Department of Cardiology, Xiangya Hospital, Central South University, Changsha, China

***Correspondence:**Jie Meng
[mengjie@csu.edu.cn](mailto:mengjie@csu.edu.cn)
Liyong Zhu
zly8128@126.com

Xiaoyun Cheng **^1,2,3^**^†^ and Zhibing Fu**^4^**^†^ These authors contributed equally to this work and share first authorship.

# Supplementary Figures

In the primary cohort, the nomogram model was internally validated using 500 bootstraps resamples, and the concordance index (C-index) was 0.871 (Supplementary Figure 1). In the internal validation, the Brier score and calibration slop were 0.154 and 0.832, respectively.

In addition, we have built (Supplementary Figure 2 and Supplementary Figure 3A) and validated another prediction model using all 93 samples (as described in Method Section). Using this new model, the AUC was 0.876 (0.809) (Supplementary Figure 3B). An evaluation of this model included a Brier score of 0.154, a calibration slope of 0.832, a C-index of 0.856 by bootstrapping (Supplementary Figure 3C), a Hosmer-Lemeshow test p-value of 0.336, and a Decision Curve Analysis (Supplementary Figure 3D). The DCA showed that the model had clinical utility when the threshold was between 0.8% and 99.8%. These performances of the new model seemed not to be more optimal than our previous model built from primary cohort, so we retained the original model.


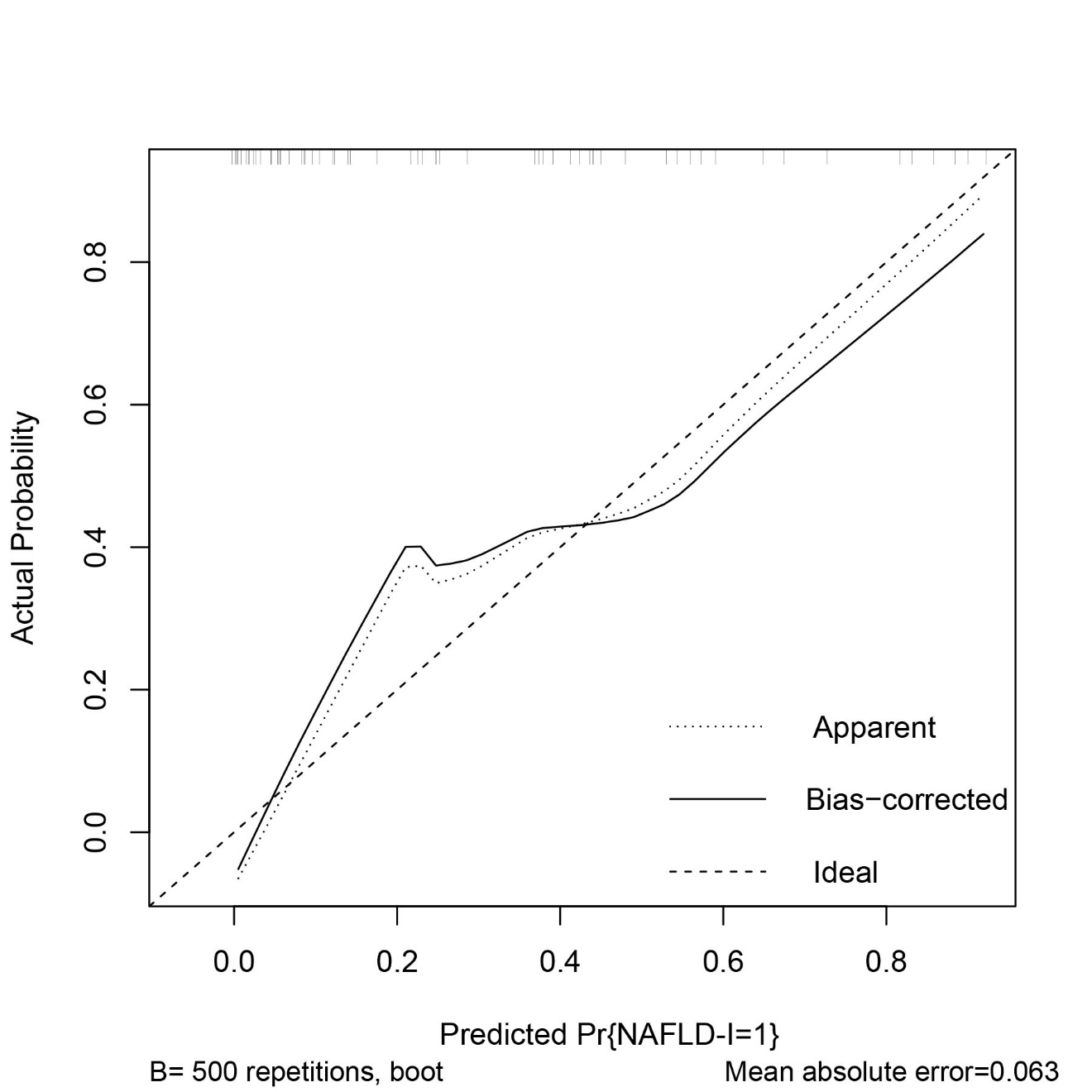


Supplementary Figure 1. A nomogram model's internal validation was conducted with 500 bootstrap resamples. NAFLD-I is an acronym for non-alcoholic fatty liver disease improvement.


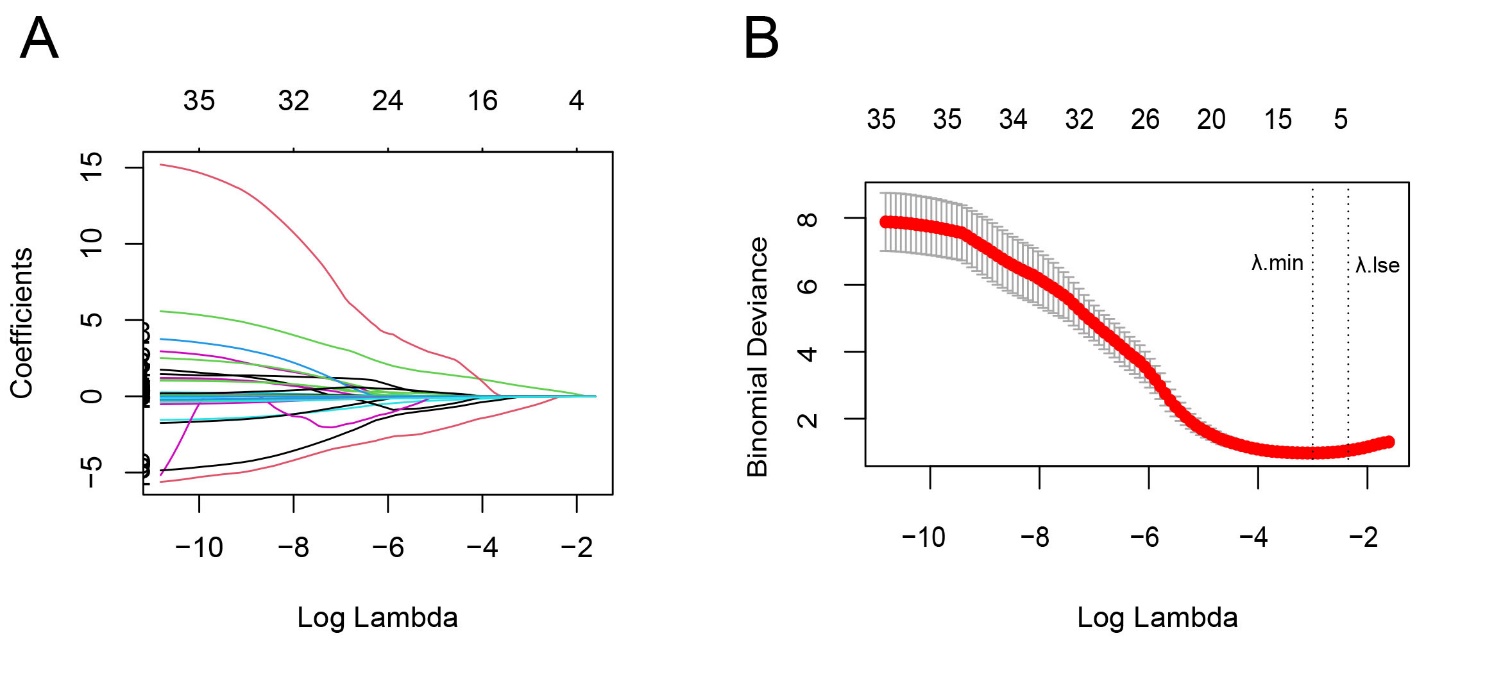


Supplementary Figure 2. Variable selection by the Lasso-Logistic regression model from all subjects (n=93). (A) Coefficient profile plotted against log(lambda). (B) Four factors with regression coefficients greater than zero were selected.


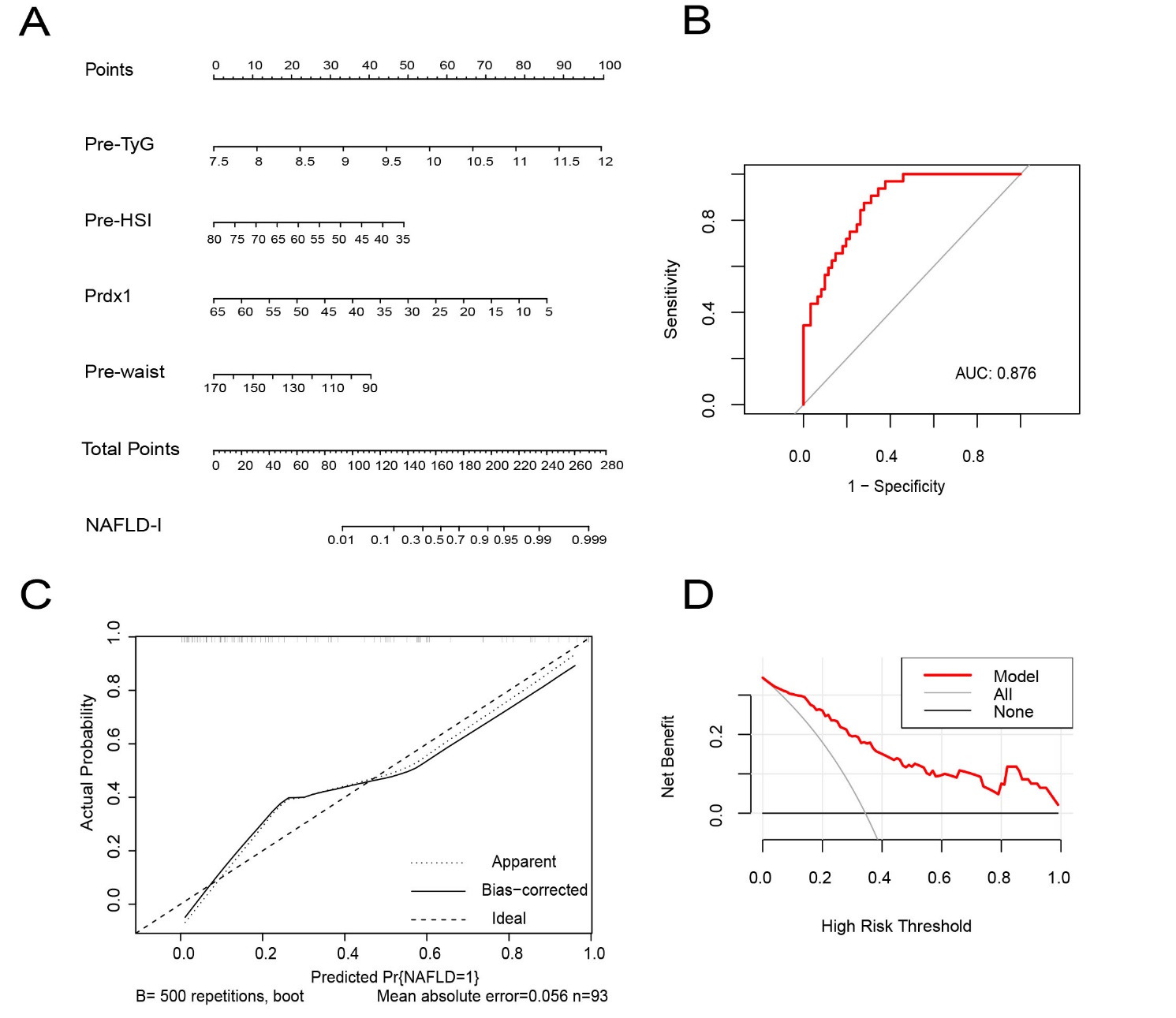


Supplementary Figure 3. Model built from all subjects (n=93).

(A) Nomogram of NAFLD improvement (NAFLD-I) at three months after LSG. (B) The ROC curves of the model. (C) Internal validation by 500 bootstrap resamples of the model. (D) decision curve analysis (DCA) of the model. The Y-axis showed net benefit. The thick solid line represented the assumption that none of NAFLD patients have been performed with LSG, while the thin solid line represented the assumption that all patients have been performed with LSG, and the red line represents that using this nomogram to predict NAFLD-I in patients.
